# Whole Genome Sequencing of Hepatitis A Virus Using a PCR-Free Single-Molecule Nanopore Sequencing Approach

**DOI:** 10.3389/fmicb.2020.00874

**Published:** 2020-05-25

**Authors:** Frederico M. Batista, Tina Stapleton, James A. Lowther, Vera G. Fonseca, Rebecca Shaw, Christopher Pond, David I. Walker, Ronny van Aerle, Jaime Martinez-Urtaza

**Affiliations:** ^1^International Centre of Excellence for Aquatic Animal Health, Centre for Environment Fisheries and Aquaculture Science (CEFAS), Weymouth, Dorset, United Kingdom; ^2^Department of Genetics and Microbiology, Facultat de Biociències – Edifici C, Campus Universitat Autònoma de Barcelona (UAB), Barcelona, Spain

**Keywords:** hepatitis A virus, *Picornaviridae*, genome, nanopore sequencing, metagenomics, SNV

## Abstract

Hepatitis A virus (HAV) is one of the most common causes of acute viral hepatitis in humans. Although HAV has a relatively small genome, there are several factors limiting whole genome sequencing such as PCR amplification artefacts and ambiguities in *de novo* assembly. The recently developed Oxford Nanopore technologies (ONT) allows single-molecule sequencing of long-size fragments of DNA or RNA using PCR-free strategies. We have sequenced the whole genome of HAV using a PCR-free approach by direct reverse-transcribed sequencing. We were able to sequence HAV cDNA and obtain reads over 7 kilobases in length containing almost the whole genome of the virus. The comparison of these raw long nanopore reads with the HAV reference wild type revealed a nucleotide sequence identity between 81.1 and 96.6%. By *de novo* assembly of all HAV reads we obtained a consensus sequence of 7362 bases, with a nucleotide sequence identity of 99.0% with the genome of the HAV strain pHM175/18f. When the assembly was performed using as reference the HAV strain pHM175/18f a consensus with a sequence similarity of 99.8 % was obtained. We have also used an ONT amplicon-based assay to sequence two fragments of the VP3 and VP1 regions which showed a sequence similarity of 100% with matching regions of the consensus sequence obtained using the direct cDNA sequencing approach. This study showed the applicability of ONT sequencing technologies to obtain the whole genome of HAV by direct cDNA nanopore sequencing, highlighting the utility of this PCR-free approach for HAV characterization and potentially other viruses of the *Picornaviridae* family.

## Introduction

Hepatitis A virus (HAV) is one of the most common causes of acute viral hepatitis in humans. According to the World Health Organization (WHO, 2017), it is estimated that HAV caused approximately 11,000 deaths worldwide in 2015. This virus is commonly transmitted through the faecal-oral route via exposure to contaminated food and water and thus considered one of the most severe food-borne viruses (Sattar et al., 2000). HAV, which belongs to the family *Picornaviridae*, is a single-stranded RNA virus with a genome of 7.5 kilobases of positive-strand polarity with a poly(A) stretch at the 3′ end (Daijogo and Semler, 2011). The virus replicates primarily in liver cells.

The development of suitable, whole-genome sequencing (WGS) methods for foodborne viruses such as HAV is key to gain a better understanding about transmission events, pathogenesis and disease-related effects (Quinones-Mateu et al., 2014). Although foodborne viruses have relatively small genomes, there are several factors limiting the sequencing of the entire genome. Some of the limitations to obtain complete genomes using high-throughput sequencing (HTS) are mainly due to the shorter reads produced. Short reads not only can impair the detection of different viral haplotypes (Takeda et al., 2019) and introduce ambiguities in *de novo* assemblies (Schatz et al., 2010) but are also unable to resolve complex regions of the genome (Somerville et al., 2019; Treangen and Salzberg, 2011). Additionally, the PCR amplification steps involved during library preparation can introduce considerable bias, for example selective amplification and chimeras (Ardui et al., 2018; Ng et al., 2018). Overall HTS can detect variant frequency but it cannot accurately map haplotype differences and consequently viral clonal populations will never be assessed completely, namely the resistant and less abundant ones (Posada-Cespedes et al., 2017).

With the advent of ‘Third generation’ sequencing approaches such as the recently developed nanopore sequencing methods by Oxford Nanopore Technologies (ONT), it is now possible to sequence single-molecules of particularly long-size fragments of DNA or RNA using amplicon-based or PCR-free strategies (Keller et al., 2018). Nanopore sequencing provides genome-length reads in real time, generating an integral vison of the genome that includes all possible mutations within a single virus particle (McNaughton et al., 2019). The particularly long reads generated by nanopore sequencing allow the complete genome to be sequenced from single viral clones and consequently make possible the detection of undescribed or rare variants (Takeda et al., 2019). The use of nanopore sequence data will increase, without precedent our ability to characterize mutant haplotypes, which reflect the immense genetic diversity of viral related sequences (aka quasispecies) within infected cells and host organisms (Andino and Domingo, 2015). This will be important to further extend our understanding of sequence variants underlying phenotype and disease. Recently nanopore sequencing was used for whole-genome sequencing of the Hepatitis C virus (Takeda et al., 2019; Ueda, 2019) and Hepatitis B virus (McNaughton et al., 2019). Nanopore sequencing was also used to serotype the Foot-and-mouth disease virus, which also belongs to the *Picornaviridae* family (Hansen et al., 2019). A major element of disease control and prevention strategies by connecting human, animal and environmental health is recognized by the One Health (OH) concept (WHO, 2017). Combining demands of OH with third generation sequencing, such as nanopore sequencing will provide unprecedent in-depth information on HAV genetic diversity in a very timely fashion. The main objective of this study was to develop a PCR-free approach using long-reads to sequence the whole genome of the hepatitis A virus.

## Materials and Methods

### Sample Preparation

The cytopathogenic pHM175 strain of HAV used in this study was originally provided in 2005 by the group of Albert Bosch at the University of Barcelona, Spain, and has since been maintained through several passages at our laboratory. HAV was cultured at 37°C in a 5% CO_2_ atmosphere in fetal rhesus kidney (FRhK-4) cell monolayers in 25 cm^2^ flasks using M2279 Minimum Essential Medium Eagle with Earle’s salts and sodium bicarbonate (1% penicillin & streptomycin, 1% 200 mM L-glutamine solution, 1% MEM non-essential amino acids (100 times), 10% for growth or 2% for maintenance of fetal bovine serum). A non-inoculated flask was used as a control. At day 12, when considerable cytopathic effects were visible, flasks were frozen at −30°C. The viral particles were then harvested by thawing the frozen flask and tapping firmly against the heel of the hand as soon as partially defrosted, to detach the cells from the flask surface. The content of the flasks was transferred to 50 mL centrifuge tubes and centrifuged for 10 min at 3000 × *g* to pellet cell debris. The supernatant was split into 1 mL aliquots, and retained at −80°C.

### HAV Quantification

A one-step quantitative reverse transcription PCR (RT-qPCR) method was used to quantify HAV where RNA from frozen FRhK cells infected with HAV was extracted as described by Tan et al. (2019). Briefly, 250 μL aliquots of viral supernatant were pipetted into 10 individual tubes containing 750 μL of TRIzol LS reagent (Invitrogen) each. After 10 min incubation at room temperature, 200 μL of chloroform was added and the mixture was shaken vigorously by hand for 30 s. This was followed by a 10 min incubation at room temperature and centrifugation at 12,000 × *g* for 15 min. The aqueous phase (colorless upper phase) was transferred into a 1.5 mL microcentrifuge tube, where equal volumes (ca. 500 μL) of isopropanol and 2 μL of glycoblue precipitant were added. The mixture was incubated overnight at −80°C. Samples were centrifuged at 12,000 × *g* for 15 min and the supernatant was discarded. RNA was washed twice with 70% ethanol and resuspended in 30 μL of nuclease-free water. For the one-Step RT-qPCR, we used the primer pair HAV68 (5′-TCA CCG CCG TTT GCC TAG-3′) and HAV240 (5′-GGA GAG CCC TGG AAG AAA G-3′) and the HAV150 MGB probe (5′-CCT GAA CCT GCA GGA ATT AA-3′) (Costafreda et al., 2006). One-Step RT-qPCR PCR reactions were performed in 25 μL final volume composed of 5 μL of RNA Ultrasense 5X Reaction Mix (Life Technologies), 1.25 μL of RNA Ultrasense enzyme mix, 0.05 μL of ROX reference dye, 0.25 μL of HAV68 primer (50 μM), 0.45 μL of HAV240 primer (50 μM), 1.25 μL of HAV150 probe (50 μM), 11.75 μL of distilled water, and 5.0 μL of template RNA. Amplification was performed by an initial incubation at 55°C for 60 min, followed by one cycle at 95°C for 5 min and 45 cycles of 15 s at 95°C, 1 min at 60°C, and 1 min at 65°C. Amplification was done on a QuantStudio 3 real-time PCR machine (Applied Biosystems). For quantification purposes, a standard curve was prepared by using serial dilutions of linear dsDNA carrying the HAV target sequence (ranging between 1x10^5^ to 1x10^1^ copies/μL).

### RNA Extraction for Sequencing

For the preparation of the sequencing libraries, RNA was extracted from FRhK cells infected with HAV using TRizol LS and the Zymo RNA clean & concentrator-5 (Zymo Research). Due to RNA limited yields we have also extracted RNA from Pacific oyster *Crassostrea gigas* gill and digestive gland tissues using the same protocol to have sufficient RNA sample to sequence using ONT. To prevent RNA shearing, samples were not vortexed throughout the whole RNA extraction procedure. Briefly, 10 separate aliquots of 250 μL of viral supernatant were added to 750 μL of TRIzol LS reagent (Invitrogen) each (a total of 2500 μL of viral supernatant was processed). Oyster RNA was chosen since oysters are a common food matrix analyzed in our lab for the detection of foodborne viruses. For oyster tissue, approximately 30 mg of gill tissue was added to 1000 μL of TRIzol LS reagent and homogenized using a disposable pestle. The mixtures were then incubated at room temperature for 10 min. In a fume hood, 200 μL of chloroform were added to each sample and samples were vigorously shaken by hand for 30 s. Following 10 min incubation at room temperature, samples were centrifuged at 12,000 × *g* for 15 min and the aqueous phase was transferred into 1.5 mL tubes. One volume of absolute ethanol was added and samples were carefully mixed by pipetting. The mixture was then processed using the Zymo RNA clean & concentrator-5 Kit as described by the manufacturer. At the final step, the RNA was eluted in 10 μL of DNase/RNase-free water. The quantity and quality of the RNA extracted from FRhK cells infected with HAV and oyster gill and digestive gland tissues were assessed using the Qubit RNA HS Assay (Invitrogen) and the Nanodrop (Thermo Fisher Scientific). The absence of HAV RNA in oyster gill and digestive gland tissues was also confirmed by RT-qPCR as described above.

### Direct cDNA Library Preparation and Nanopore Sequencing

Library preparation was carried out using a direct cDNA native barcoding protocol (SQK-DCS 109 with EXP-NBD104) as recommended by ONT (Version: DCB_9091_v109_revH_14Aug2019). The amount of RNA recommended by ONT for this protocol is 100 ng of polyA+RNA. Two samples were prepared using: 1) a mixture of RNA extracted from FRhK cells infected with HAV and RNA extracted from oyster tissue (HAV + oyster); and 2) RNA extracted from oyster tissues (oyster). For the preparation of the first library, 49 ng of RNA extracted from FRhK cells infected with HAV was mixed with 51 ng of oyster tissue RNA. For the second library 100 ng of oyster tissue RNA was used. For library preparation, the first step started with cDNA synthesis using reverse transcription and strand-switching. Briefly, 100 ng of RNA was resuspended in 7.5 μl of RNase-free water and mixed with 2.5 μl of the VN primer (ONT; 2 μM) that targets the poly-A tail and 1 μl of dNTPs (10 mM total concentration). The solution was mixed by gently flicking the tube, incubated for 5 min at 65°C and immediately snap cooled on a freezer block. In a separate tube the following reaction mix was prepared: 4 μl of 5X RT buffer (ONT), 1 μl of RNaseOUT (40 U/μl, Life technologies), 2 μl of strand-switching Primer (10 μM, ONT) and 1 μl of RNase-free water. The mixture was mixed by gently flicking the tube then added to the snap-cooled RNA prepared above and incubated for 2 min at 42°C. One μl of Maxima H Minus Reverse transcriptase (Life Technologies) was added and the mixture was incubated for 90 min at 42°C followed by heat inactivation at 85°C for 5 min and finally held at 4°C. RNA was then degraded by adding 1 μl of RNase Cocktail Enzyme Mix (Thermo Fisher) followed by incubation at 37°C for 10 min. The cDNA was then purified using AMPure XP beads as described in the ONT protocol and eluted in 20 μl of nuclease-free water. The second strand was synthetized in a 50 μl reaction composed of 25 μl of 2x LongAmp Taq Master mix (New England Biolabs), 2 μl of PR2 primer (ONT), 3 μl of nuclease free-water and 20 μl of reverse-transcribed sample from above. The mixture was incubated at 94°C for 1 min, 50°C for 1 min, 65°C for 15 min and hold at 4°C. The cDNA was then purified using AMPure XP beads as described in the ONT protocol and eluted in 21 μl of nuclease-free water. In the final step of the library preparation, the ends of the cDNA fragment were repaired to create blunt ends and dA-tails were added. This was performed by mixing the following reagents in a 0.2 ml PCR tube: 20 μl of the cDNA prepared above, 30 μl of nuclease-free water, 7 μl of Ultra II End-prep reaction buffer (New England Biolabs) and 3 μl of Ultra II End-prep mix (New England Biolabs). After gentle mixing by pipetting, the reaction mix was incubated in a thermal cycler for 5 min at 20°C then 5 min at 65°C. The cDNA was then purified using AMPure XP beads as described in the ONT protocol and resuspended in 22.5 μl of nuclease-free water. Sample barcoding was carried out using the Native Barcoding Expansion kit (EXP-NBD104), according to manufacturer’s instruction. Here, the ‘HAV + oyster’ sample was barcoded with the NB01 barcode and the ‘oyster’ sample with NB02. Barcoding was carried out in a 50 μl reaction with 22.5 μl of end-prep cDNA, 2.5 μl of native barcode (NB01 or NB02) and 25 μl of Blunt/TA ligase Master Mix (New England Biolabs). After mixing by flicking the tube, the reaction mix was incubated for 10 min at room temperature. The barcoded cDNA was then purified using AMPure XP beads as described in the ONT protocol and resuspended in 26 μl of nuclease-free water. The barcoded samples were pooled, and the volume adjusted to 65 μl to which 5 μl of Adapter Mix II (AMII, ONT), 20 μl of 5X NEBNext Quick Ligation Reaction Buffer (New England Biolabs) and 10 μl of Quick T4 DNA ligase (New England Biolabs) were added. The reaction mix was incubated at room temperature for 10 min and purified using AMPure XP beads as described in the ONT protocol and resuspended in 13 μl of nuclease-free water. 12 μl of the library solution were mixed with 37.5 μl of sequencing buffer (SQB, ONT) and 25.5 μl of Loading Beads (LB, ONT) and loaded into a flow cell (FLO-MIN106, ONT) equipped with R9.4.1 chemistry on a MinION (Mk1B, ONT) device. The MinION was operated using MinKNOW and the flow cell was primed following manufacturer’s instructions, with a total run time of 26 h.

### Amplicon Library Preparation and Nanopore Sequencing

**FIGURE 1:**
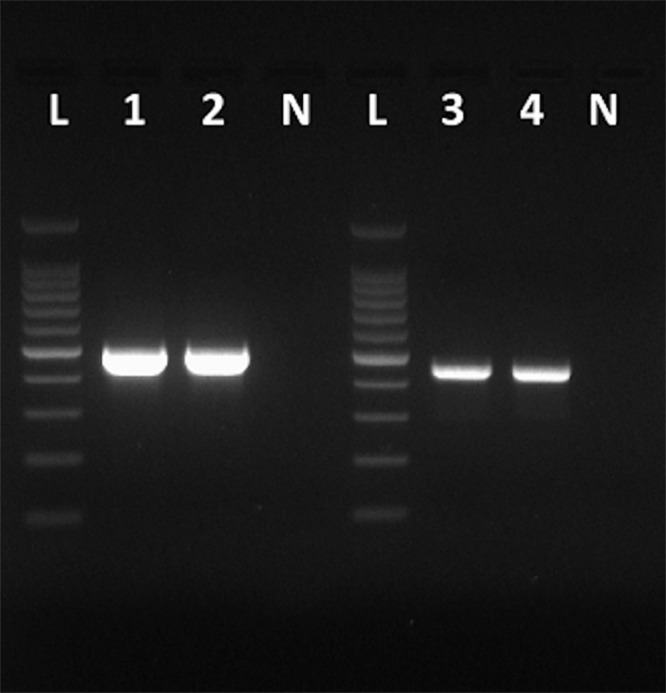
Amplicons obtained with the primers VP1F and VP1R that amplify a 498-bp fragment of the VP1 region of HAV (Lanes 1 and 2), and amplicons obtained with the primers NH_2_ and VP3-1431B that amplify a 455-bp fragment of the VP3 region of HAV (Lanes 3 and 4). Lanes N, negative controls. Lanes L, 100 bp DNA ladder (Promega).

For the amplicon library preparation, cDNA was synthetized using RNA extracted from HAV FRhK infected cells as described above. For the reverse transcription, 10 μl of RNA, 1 μl of random hexamers (50 μM, Sigma), 1 μl of dNTPs (10 mM, New England Biolabs) and 1 μl of nuclease-free water were mixed and incubated at 65 °C for 5 min and immediately after placed on ice for 4 min. In a separate tube, a mixture was prepared containing 4 μl of SuperScript IV RT buffer (Invitrogen, Life Technologies), 1 μl of DTT (1 mM, Invitrogen, Life Technologies), 1 μl of RNaseOUT (40 U/μl, Invitrogen, Life Technologies) and 1 μl of SuperScript IV (200 U/μl, Invitrogen, Life Technologies). This mixture was added to the RNA-primer mix and incubated at 23°C for 10 min followed by 30 min at 50°C and inactivation of the reaction at 80°C for 10 min. A 455-bp fragment of VP3 region (corresponding to nucleotides 1470 to 1839 of HAV M59808) was amplified by PCR using the forward primer VP3-1431B (5′-CACT CAA TGT TTT AGC TAG A-3′) and reverse primer NH_2_-VP3 (5′- TCT ACC TGA ATG ATA TTT GG -3′) described by Sanchez et al. (2003). Additionally, a 498 bp fragment of the VP1 region (corresponding to nucleotides 2394 to 2891 of HAV M59808) was amplified by PCR using the forward VP1F primer (5′- TCC TGA ATT GAA ACC TGG AGA A -3′) and the reverse VP1R_primer (5′- GCA ATC TGA ATG AAA CCA ATC CA -3′), designed using primer3 software with default parameters and the consensus obtained in the present study (by directed cDNA sequencing) as the source sequence. For both VP1 and VP3 fragments amplification, PCR reactions were performed in a final reaction mix volume of 25 μl and contained 5 μl of 5X Colorless GoTaq Flexi Buffer (Promega), 12.3 μl of nuclease free water, 1 μl of dNTP (10 mM, New England Biolabs), 2 μl of MgCl_2_ (25 mM, Promega), 1.25 μl of each primer (10 μM), 0.2 μl of GoTaq G2 Hot Start (Promega) and 2.0 μl of template cDNA. The amplification cycle consisted of an initial denaturation step at 95°C for 2 min, followed by 38 cycles of 30 s at 95°C, 30 s at 50°C, 1 min at 72°C and with a final extension of 10 min at 72°C. All PCR reactions were performed in an Eppendorf Master Cycler Nexus X2, (Eppendorf). Amplicons were separated by electrophoresis on 1.5% agarose gels, stained with Ethidium bromide and visualized under UV light to confirm the expected size and absence of non-specific amplification (Figure 1). The quantity of the amplicons obtained was determined using the Qubit DNA HS Assay (Invitrogen, Life Technologies). Library preparation was carried out using an Amplicon by ligation protocol (SQK-LSK109) recommended by ONT (Version: DGE_9063_v109_revS_14Aug2019) but using a Flongle device (flow cell and adapter). Amplicons were repaired and end-prepped by mixing 0.5 μl DNA CS (ONT), 23.5 μl of amplicons (ca. 140 fmol), 1.75 μl NEBNext FFPE DNA Repair Buffer (New England Biolabs), 1 μl NEBNext FFPE DNA Repair Mix (New Englands Biolabs), 1.75 μl Ultra II End-prep reaction buffer (New England Biolabs) and 1.5 μl Ultra II End-prep enzyme mix (New England Biolabs). The mixture was mixed by gently flicking the tube, spin down and incubated at 20 °C for 5 min and 65°C for 5 min. The amplicons were cleaned-up using AMPure XP beads as described in the ONT protocol and eluted in 31 μl of nuclease-free water. Adapters were then ligated by mixing 30 μl of amplicons prepared in the previous step, 12.5 μl of ligation buffer (ONT), 5 μl of NEBNext Quick T4 DNA ligase (New England Biolabs) and 2.5 μl of adapter mix (ONT). The mixture was mixed by gently flicking the tube, spin down and incubated at room temperature for 10 min. The amplicons were cleaned-up using AMPure XP beads and using 125 μl of fragment buffer (ONT) as described in the ONT protocol and the pellet was resuspended in 8 μl of elution buffer (ONT). Three μl of this suspension were used for quantification using the Qubit DNA HS Assay (Invitrogen, Life Technologies). The sequencing mix was prepared using 10 fmol of each prepared sample (VP1 or VP3) and eluted in elution buffer (ONT) in a total volume of 5 μl which was then mixed with 10 μl of loading beads (ONT) and 15 μl of sequencing buffer (ONT). The Flongle flow cell (which had 71 active nanopores) was then primed with a mix of 117 μl of flush buffer with 3 μl of flush tether, followed by the addition of 30 μl of the sequencing mix. The Flongle was operated using MinKNOW and the total run time was 16 h.

### Bioinformatic Analysis

Base calling of Fast5 files was performed in a MinIT (ONT-minit-release 19.05.2) with Flip Flop algorithm. Demultiplexing and removal of barcode sequences was performed using Porechop (v0.2.3, https://github.com/rrwick/Porechop). Since a large proportion of the reads were not successfully barcoded, all reads were pooled for further analysis. Run metrics were calculated using the Nanoplot (v1.20.0) and reads were filtered (sequence quality > 7) using Nanofilt (v2.3.0) (De Coster et al., 2018). Filtered fastq files were converted to fasta files, which were then used to create a cDNA nucleotide database using Blast v2.9.0+ (Camacho et al., 2009). We used as query a hepatitis A virus genome (strain HM-175, wild type, Genbank accession number M14707) which was blasted against our cDNA nucleotide database to detect HAV reads. Reads showing a significant e-value were extracted from the original fasta files and screened for chimeras using YACRD, which resulted in 302 HAV chimera-free reads (Marijon et al., 2019). The extracted HAV reads were mapped against the HAV genome (Genbank accession number M14707) using Minimap2 (Li, 2018) and the alignment was visualized using Tablet (Milne et al., 2013). The HAV chimera-free 302 reads were assembled using Canu (v1.8) using default parameters for nanopore sequence data (Koren et al., 2017) to produce a consensus nucleotide sequence. Moreover, a consensus sequence was obtained by mapping the HAV reads to HAV cytopathogenic pHM175/18f strain genome (accession number M59808) using LAST sequence aligner (version 923, with default parameters) as implemented in Nanopipe (Shabardina et al., 2019). Multiple alignments were carried out using MAFFT v7 software (Katoh and Standley, 2013) using default parameters, with the nucleotide sequences of the consensus obtained in the present study using Canu and the genomes of HAV genotypes IA (KC182590, EF406357, X75215, X83303), IB (AF314208, M20273, M14707, AF268396, M59808), IIA (AY644676), IIB(AY644670), IIIA (AJ299464), IIIB (AB258387) as well as the simian HAV genome (EU140838). The sequence alignment region used for downstream analysis, was retained from position 84 up to 7422 regions of the HAV genome M14707. Maximum Likelihood (ML) analysis was conducted on the sequence alignment region shared by all genome sequences using MEGA7 (Kumar et al., 2016) using the General Time Reversible model (Nei and Kumar, 2000), gamma distribution with invariant sites (G+I), four discrete rate categories and 1,000 bootstrap replicates. Single nucleotide variants (SNVs) were called using Longshot software with a strand bias p-value of 0.01 (-c 50, -C 100,000) (Edge and Bansal, 2019).

## Results and Discussion

### RNA Extraction and Purification

An important factor that impacts cDNA synthesis from RNA viruses and ONT library preparation is the quality of the RNA and the absence of contaminants that can be carried over during the RNA extraction. In our experiments, these parameters could not be fully explored using the nanodrop OD 260/280 and 260/230 ratios for the FRhK cells/viral RNA due to the low concentration obtained in our samples (7 ng/μL, Qubit quantification). For oyster RNA (1562 ng/μL, Qubit quantification), the nanodrop OD 260/280 and 260/230 ratios were 2.11 and 2.19, respectively. These values are indicative of pure RNA with no indication of contaminants. We have tested other RNA extraction and purification methods (data not shown) but these have often resulted in lower 260/230 ratios comparatively to the TRIzol/Zymo kit used. Other methods were therefore not used for RNA preparation in this work. The HAV level quantified by RT-qPCR was 1.7 × 10^8^ copies/mL of cell-culture supernatant fluid. The recommended quantity of RNA for the direct cDNA ONT protocol (SQK-DCS109) is 100 ng of RNA with a polyA tail. As the total RNA extracted from FRhK cells infected with HAV for library preparation was below this value (i.e., a total of 49 ng obtained from 2.5 ml of viral suspension) an additional 51 ng of oyster RNA was added. Based on the RT-qPCR results, we estimated that ca. 4 × 10^8^ viral genome copies were used for the library preparation which should equate to approximately 1.81 ng of viral RNA (0.71 fmol) assuming a genome size of 7.5 kilobases. Therefore, the estimated amount of eukaryotic RNA extracted from the FRhK cells and oyster gills was 288.4 fmol and hence 406 times more than viral RNA copies. Most importantly, such limited amount of viral RNA often represents a limiting factor for RNA or cDNA direct sequencing approaches (Posada-Cespedes et al., 2017). In clinical and food samples PCR-based methods are currently the only possible approach to sequence viral RNA (Acevedo and Andino, 2014). Regardless, whole-genome sequencing using PCR-based approaches can also be quite laborious and are known to induce several PCR amplification artifacts and bias (Ardui et al., 2018).

### Direct cDNA Nanopore Sequencing

**FIGURE 2:**
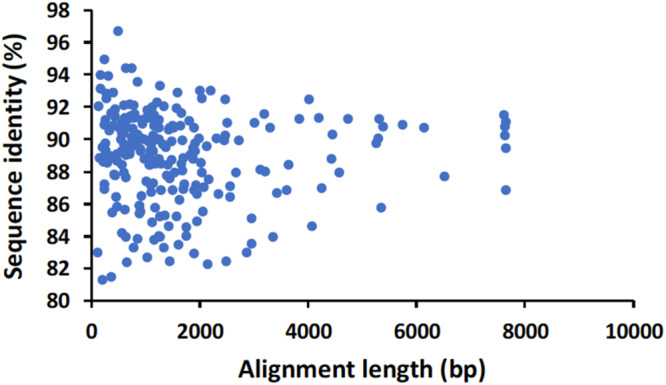
Alignment length and nucleotide sequence identity of raw nanopore hepatitis A virus reads identified by BLASTn analysis using the genome of HAV wild type (M14707) as a reference.

**FIGURE 3:**
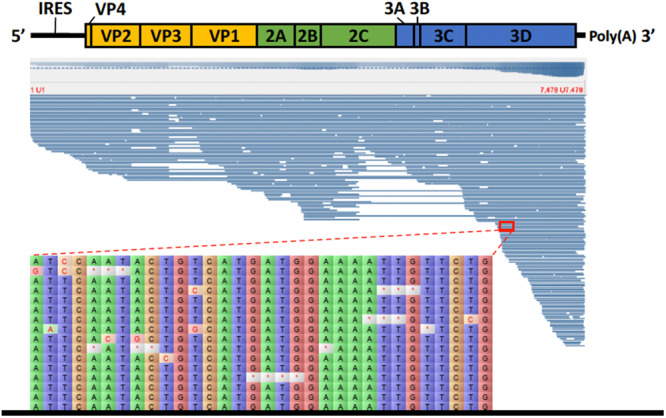
Alignment of HAV nanopore reads obtained by direct cDNA sequencing using the genome of HAV wild type (M14707) as a reference. Detail of part of the alignment of 16 HAV nanopore reads highlighting the deletions (marked with a red asterisk) and substitutions.

**FIGURE 4:**
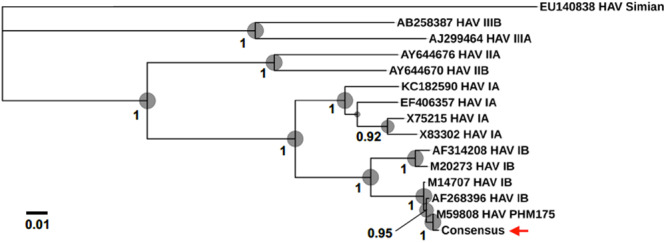
Phylogenomic analysis using HAV genomes (several Human genotypes IA, IB, IIA, IIB, IIIA, and IIIB as well as a Simian HAV) and the consensus sequence obtained in the present study (marked with a red arrow). Bootstrap values obtained by Maximum Likelihood analysis are indicated at the main nodes.

For the direct cDNA approach, a total of 2,792,673 nanopore raw reads were obtained with a N50 (i.e., defined as the minimum contig length needed to cover 50% of the genome) of 1,044 bp and mean read length of 946 bp. The mean read quality of unfiltered reads was 7.5 and approximately 67% of the reads had a quality above 7 (Q7). After eliminating reads with a quality below 7 a total of 1,860,348 reads were retained. A BLASTn search using the HAV genome (M14707) as the query and the Q7 reads as the database allowed the identification of 243 HAV reads (before chimera analysis) with a significant e-value (<1E-23). The percentage of HAV reads relative to the total number of reads was only 0.013% which is considerably less than the expected 0.245% based on the estimated quantification by RT-qPCR described above. The current protocol also required the presence of a poly A-tailed RNA and thus cDNA will only be synthesized for RNA containing a poly A tail, which in HAV it stretches at the 3′ end (Daijogo and Semler, 2011). The relatively low number of HAV reads detected can be partially explained by RNA shearing during extraction and library preparation. Notwithstanding, several long sequence reads (with more than 7Kb) representing HAV sequences were identified, suggesting that some viral particles were still intact. The sequence identity of the HAV reads ranged between 81.1 and 96.6% with a mean value of 88.9% and a standard deviation of 2.8%, when using the HAV wild type genome (M14707) as a reference (Figure 2). The length of these reads ranged between 129 and 7667 bases with a median value of 1173 bases. When analyzing only the long reads that had more than 7.3 kilobases, these contained almost the whole genome of the HAV. In fact, when comparing the 7.3 Kb reads with the HAV reference wild type (M14707) the nucleotide sequence identity was between 84.7 and 90.6% with indels and substitutions accounting for a mean value of 5.6 and 5.9% of the dissimilarity, respectively. The resulting reference-based mapping covered the whole genome of the HAV (Figure 3). One of the major limitations of nanopore sequencing is still its relatively high error rates, with the proportion of read errors often ranging from 10-30% (Goodwin et al., 2015). Nonetheless several algorithms have been developed to reduce these sequencing errors (Goodwin et al., 2015; Salmela et al., 2017). In order to circumvent reference-based read alignment biases, we then utilized the Canu software that operates by improving the accuracy of bases in reads, trim reads to the portion that have high-quality sequence followed by *de novo* assembly. From this *de novo* assembly a consensus nucleotide sequence with 7362 bases was obtained (after trimming the poly A tail and the 3′-end using the M14707 HAV genome as reference). This consensus showed a nucleotide sequence identity of 99.0% (7349 out of 7423 identical bases) against the genome of a HAV cytopathogenic pHM175/18f strain (accession number M59808). We identified 61 deletions and 13 substitutions along the 7423 bases alignment of the consensus with the HAV cytopathogenic pHM175/18f strain (M59808). Phylogenetic analysis of the HAV consensus obtained using Canu and reference sequences of known genotypes showed that the consensus clustered robustly with the sub-genotype IB group (Figure 4). However, when the assembly was performed using the HAV strain pHM175/18f (M59808) as reference a consensus with a sequence similarity of 99.8 % was obtained (7423 bases, Genbank accession number MT181522). The alignment of this reference-based consensus with HAV M59808 strain revealed 12 substitutions and 1 ambiguous position. Since no other sequencing methods (e.g., Illumina sequencing, which has a much lower error rate) were used in this study, it was not possible to determine the sequencing errors accurately. Nevertheless, the 0.2% nucleotide sequence dissimilarity observed between the consensus and the HAV cytopathogenic pHM175/18f strain suggests that it is possible to circumvent ONT sequencing errors by using fine-tuned data analysis pipelines. Notwithstanding, RNA viruses exhibit high levels of mutation rates (Duffy et al., 2008) and this can also contribute to the presence of new viral strains during replication via point mutations, insertions and deletions (Posada-Cespedes et al., 2017), especially in strains used in laboratories after many passages and cultivation.

### HAV Genetic Diversity

**FIGURE 5:**
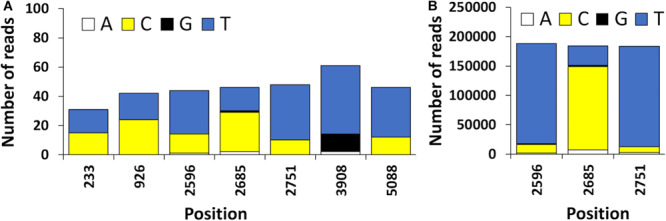
**(A)** Position and frequency of nucleotides of candidate sequence nucleotide variants (SNVs) detected using the HAV reads obtained by ONT direct cDNA sequencing. **(B)** Frequency of nucleotides observed in the VP1 amplicon dataset. The position of the nucleotides is shown using the HAV cytopathogenic pHM175/18f strain genome as reference (accession number M59808).

The high error rate of nanopore sequencing makes the detection of single nucleotide variants quite challenging (Vasudevan et al., 2019). These technical limitations present in Oxford nanopore sequence data can be bypassed with bioinformatic tools that enable to distinguish artifacts from true polymorphisms, such is the case of the newly developed Longshot software (Edge and Bansal, 2019). In the present study, 7 possible SNVs were detected using Longshot software being 6 transitions and 1 transversion (Figure 5). Previous studies have also reported a considerably higher number of transitions over transversions in HAV (Sanchez et al., 2003). These results suggest that the HAV population characterized might have been composed by two or more haplotypes. This highlights the potential of direct cDNA nanopore sequencing to characterize the genetic diversity of RNA viruses, which can be an alternative to PCR based HTS methods and its inherent amplification biases (Posada-Cespedes et al., 2017). To confirm the presence of SNVs detected using direct cDNA sequencing, we used an ONT amplicon-based approach targeting two regions of the HAV genome (VP1 and VP3). For the VP3 amplicon dataset, a total of 192, 738 reads were mapped against HAV M59808 strain. The 452-bp consensus sequence obtained (without the annealing regions) showed 100% nucleotide sequence similarity with the consensus obtained using the cDNA approach. Moreover, no polymorphisms were detected in the VP3 amplicon dataset which is in accordance with the results obtained using the direct cDNA dataset. For the VP1 amplicon dataset, a total of 124,986 reads were mapped against the HAV M59808 strain. One hundred percent nucleotide sequence similarity was also observed between the 415-bp consensus sequence obtained using the amplicon data and the consensus obtained using the direct cDNA sequencing approach. However, no polymorphisms were detected in the VP1 amplicon dataset which contrasts with the 3 SNVs detected in the same region using the direct cDNA sequencing approach (positions 2596, 2685, and 2751, Figure 5). This could be due to the fact that amplification steps can exponentially amplify the most abundant viral clones, resulting in scarce or absent representation of low abundance viral haplotypes (Posada-Cespedes et al., 2017). In this regard, our PCR-free approach could circumvent these amplification bias.

## Conclusion

In the present study we demonstrated the potential of nanopore single-molecule sequencing to sequence the whole genome of HAV from infected cell lines. We were able to sequence HAV by direct cDNA sequencing using a PCR-free nanopore sequencing approach and obtained long-reads some of which were over 7.3 kilobases in length, containing nearly the whole genome of the virus.

## Data Availability Statement

The datasets generated for this study can be found in Genbank, under accession number MT181522.

## Author Contributions

FB and JM-U conceived and designed the study. FB, TS, JL, RS, and CP did the laboratory work. FB, VF, and RvA did the bioinformatic analyses. FB, TS, JL, VF, CP, DW, RvA, and JM-U wrote the manuscript.

## Conflict of Interest

The authors declare that the research was conducted in the absence of any commercial or financial relationships that could be construed as a potential conflict of interest.
